# Social Saliency of the Cue Slows Attention Shifts

**DOI:** 10.3389/fpsyg.2017.00738

**Published:** 2017-05-15

**Authors:** Vassiki Chauhan, Matteo Visconti di Oleggio Castello, Alireza Soltani, Maria Ida Gobbini

**Affiliations:** ^1^Department of Psychological and Brain Sciences, Dartmouth College, HanoverNH, USA; ^2^Dipartimento di Medicina Specialistica, Diagnostica e Sperimentale, Medical School, University of BolognaBologna, Italy

**Keywords:** personal familiarity, cue salience, social cues, gaze cueing, eye gaze, face processing, slowed disengagement of attention

## Abstract

Eye gaze is a powerful cue that indicates where another person’s attention is directed in the environment. Seeing another person’s eye gaze shift spontaneously and reflexively elicits a shift of one’s own attention to the same region in space. Here, we investigated whether reallocation of attention in the direction of eye gaze is modulated by personal familiarity with faces. On the one hand, the eye gaze of a close friend should be more effective in redirecting our attention as compared to the eye gaze of a stranger. On the other hand, the social relevance of a familiar face might itself hold attention and, thereby, slow lateral shifts of attention. To distinguish between these possibilities, we measured the efficacy of the eye gaze of personally familiar and unfamiliar faces as directional attention cues using adapted versions of the Posner paradigm with saccadic and manual responses. We found that attention shifts were slower when elicited by a perceived change in the eye gaze of a familiar individual as compared to attention shifts elicited by unfamiliar faces at short latencies (100 ms). We also measured simple detection of change in direction of gaze in personally familiar and unfamiliar faces to test whether slower attention shifts were due to slower detection. Participants detected changes in eye gaze faster for familiar faces than for unfamiliar faces. Our results suggest that personally familiar faces briefly hold attention due to their social relevance, thereby slowing shifts of attention, even though the direction of eye movements are detected faster in familiar faces.

## Introduction

Social cues, such as direction of eye gaze and head angle, are effective in redirecting one’s attention to salient aspects of the environment (Friesen and Kingstone, 1998; Driver et al., 1999; Langton and Bruce, 1999; Hoffman and Haxby, 2000; Pelphrey et al., 2003; Senju and Csibra, 2008; Senju and Johnson, 2009). Here, we investigated whether reallocation of spatial attention was faster in response to the shift in eye gaze of a familiar individual as compared to the shift in eye gaze of a stranger.

Several studies have demonstrated that perceived eye gaze spontaneously biases spatial attention in the direction of the gaze (Friesen and Kingstone, 1998; Driver et al., 1999; Langton and Bruce, 1999; Hoffman and Haxby, 2000; Pelphrey et al., 2005; Frischen et al., 2007; Senju and Csibra, 2008; Senju and Johnson, 2009). These biases in spatial attention have previously been shown to be susceptible to top-down influences (Teufel et al., 2010; Kawai, 2011; Wiese et al., 2012, 2014; Wykowska et al., 2014). The neural mechanisms underlying gaze perception have been studied extensively. fMRI studies have shown that specific regions, such as the posterior and anterior superior temporal sulcus, the intraparietal sulcus, the medial prefrontal cortex, are preferentially engaged by eye gaze and head angles, highlighting how dedicated populations of neurons are involved in processing these social cues (Hoffman and Haxby, 2000; Pageler et al., 2003; Pelphrey et al., 2003; Engell and Haxby, 2007; Carlin et al., 2012; Carlin and Calder, 2013; and for a review, Senju and Johnson, 2009). Interestingly, eye gaze and head position are processed even without awareness, underscoring that detection of these features, and the subsequent effect on spatial attention, can be preconscious and any facilitation or slowing by familiarity may be acting upon a very early stage of processing (Stein et al., 2011; Gobbini et al., 2013a).

Personally familiar faces are highly salient social stimuli (Gobbini and Haxby, 2007). Efficient face processing through multimodal experience with familiar others serves to facilitate interactions with those who are most important in our social lives. Familiarity affords more efficient face detection and recognition of identity (Bruce et al., 1994; Burton et al., 1999; Jenkins et al., 2011; Ramon et al., 2011; Gobbini et al., 2013b; Ramon, 2015; Visconti di Oleggio Castello and Gobbini, 2015; Ramon and Van Belle, 2016; Visconti di Oleggio Castello et al., 2016). Similarly, processing of social cues in faces, such as direction of attention, are markedly facilitated by familiarity. For example, in a study of perception of gaze direction and head angle, perception of eye gaze was detected around 100 ms faster in familiar as compared to the faces of strangers (Visconti di Oleggio Castello et al., 2014).

Here, we investigated whether personal familiarity with the faces whose change in eye gaze served as attentional cue has an influence on how we reallocate spatial attention in response to those eye gaze changes. We used a gaze cueing paradigm – an adaptation of the Posner cueing paradigm (Posner, 1980). In the Posner cueing paradigm, presentation of a directional cue (e.g., an arrow) precedes the onset of a target in either the cued or uncued direction. Participants are faster and more accurate in processing a target if it appears in the cued direction (Posner, 1980). In the gaze cueing paradigm, faces with directional eye gaze served as the directional cue for manipulating spatial attention. Perceived eye gaze has proved successful in modulating spatial attention in schematic faces (Friesen and Kingstone, 1998). One could hypothesize that facilitation of detection of eye gaze direction in personally familiar faces leads to more effective redirection of spatial attention as compared to detection of eye gaze shifts in the faces of strangers (Visconti di Oleggio Castello et al., 2014). As an alternative hypothesis, highly salient faces of familiar individuals could hold attention (Bindemann et al., 2005), thus making redirecting spatial attention slower, similar to a highly rewarding feature in visual search tasks (Hickey et al., 2010; Anderson et al., 2011; Hickey and Peelen, 2015; Munneke et al., 2015). Allocation of attentional resources is driven by relative salience of stimuli encountered in the environment, where high salience can be generated by the relative configuration of low-level features such as color, orientation and size in the scene (Itti and Koch, 2001; Soltani and Koch, 2010) or by an interaction between sensory features and reward (Markowitz et al., 2011; Theeuwes and Belopolsky, 2012; Khorsand et al., 2015). Similarly, valence of the face cue (e.g., faces with expression of emotion such as fear, happy, neutral, or sad or one’s own face; Pourtois et al., 2013; Pérez-Duenas et al., 2014; Porciello et al., 2014) has been shown to modulate the magnitude of attentional capture. Thus, spatial reallocation of attention in response to gaze shifts in familiar faces could be facilitated by faster processing of the gaze cue or slowed by the social saliency of familiar faces.

We tested these two competing hypotheses on how face familiarity interacts with redirection of attention in response to shifts in eye gaze with two experiments. In the first experiment, we asked participants to make a saccade to the target appearing in the periphery either on the right or on the left of a centrally presented face. In the second experiment, we asked the participants to report the side on which the target appeared with a button press, without looking away from the centrally presented face. We used the manual response paradigm to test whether slowed initiation of saccades to a target is attributable to the difficulty of breaking fixation from salient centrally presented faces. Finally, we performed a control experiment to ensure that results from the first two experiments on reallocation of attention were not due to differences in detecting eye movements in personally familiar and unfamiliar faces (Visconti di Oleggio Castello et al., 2014). In this experiment, we measured participants’ speed in detecting a change in the direction of eye gaze in personally familiar and unfamiliar faces.

## Experiment 1

In Experiment 1, we investigated attention shifts elicited by the eye gaze of familiar and unfamiliar faces using a target-detection task based on the Posner cueing paradigm, with saccadic reaction time (SRT) as the dependent variable. Participants saw a directional gaze cue to the left or right in a familiar or unfamiliar face followed by a peripheral target that could appear on either side of the fixation cross. They were instructed to saccade toward the target as soon as it appeared on the screen. We manipulated the familiarity of the face cue, the congruence between the cue and target direction, and the delay between the cue and the target onset. Participants were instructed to be as fast as possible in their response, but not at the expense of accuracy.

### Materials and Methods

#### Participants

Fifteen students from the Dartmouth College community participated in Experiment 1 (seven male, Mean age: 26.4 ± 3.22). All participants were right handed with the exception of one. All participants provided written informed consent to participate in the experiment, and were paid. The Dartmouth Committee for the Protection of Human Subjects approved the experiment (Protocol 21200).

The number of participants was determined with a power analysis performed with the package ‘pwr’ in R (Champely, 2009) to ensure that the sample size was large enough to replicate the effect of gaze cueing reported previously (Kuhn and Kingstone, 2009). Other studies exploring the effects of gaze cueing have used similar sample sizes (Ricciardelli et al., 2002; Kuhn and Kingstone, 2009; Hungr and Hunt, 2012).

#### Equipment

All stimuli were presented on an FSI AM250 monitor, which has a refresh rate of 60 Hz. The resolution of the display was 1920 × 1080 pixels. Eye movement data was collected with an Eyelink 1000 Plus Desktop Mount eye tracker system. Participants were seated 60 cm from the presentation screen throughout the course of the experiment, with their chin on a chin rest to minimize head movements.

#### Stimuli

Grayscale pictures of friends of the participants were used as familiar stimuli. All friends were students from the Dartmouth Community with whom participants had a good relationship for at least 1 year. Unfamiliar stimuli were pictures of age and gender matched controls that were taken at another college in an identical studio setting, using the same lighting and camera, to guarantee equivalent picture quality. For each participant, we used pictures of three friends and three strangers.

We used the following procedure to construct the face cue stimuli. We used a full face image with direct eye gaze of each identity as the base image, then superimposed an image of the pupil and iris from images of the same identity looking to the left or right. Minor smoothing with GIMP was performed to give the images a natural appearance. Thus, three images were constructed for each identity: eyes gazing forward, eyes gazing to the left, and eyes gazing to the right.

To avoid confounds due to low-level visual properties of stimuli, all the stimulus images were matched to the average luminance value of all the pictures and for contrast with the lumMatch function from the SHINE toolbox (Willenbockel et al., 2010).

#### Experimental Paradigm

Experiment 1 consisted of five blocks of 120 trials each, resulting in a total of 600 trials in the entire experiment. The experimental manipulations were Familiarity of the faces (Familiar or Unfamiliar), Validity of Cue (Congruent or Incongruent), and stimulus onset asynchrony (SOA) between the cue and target (100 or 200 ms). These SOAs cover the initial stages of the effect of attention reallocation when it is building up and reaching its maximum. All three conditions were counterbalanced. Over the entire experiment, the picture of each individual identity was repeated 100 times. Version 3.0.12 of Psychtoolbox (Kleiner et al., 2007) on MATLAB 2014b was used for the purpose of stimulus presentation and response collection.

At the beginning of each block the eye-tracker was calibrated and validated with a five-point calibration. The trial started with a centrally presented black fixation cross. The fixation cross subtended 1 × 1 degree of visual angle around the center of the screen. Each trial started with a button press by the participant. Following the button press, the fixation cross was replaced by the image of a face gazing forward. Each face image subtended 3 × 4.3 degrees of visual angle centered on the center of the screen. The forward gazing face remained onscreen for a jittered interval between 750 and 1000 ms, in order to avoid a build-up of expectation for the gaze cue. This was followed by a gaze shift to the left or right, and after an SOA of either 100 or 200 ms, a black target circle appeared in either the cued or uncued location. The gaze cue was valid for half the trials in each block and, thus, was uninformative. Others have shown (e.g., Friesen and Kingstone, 1998) that gaze cues do not have to be informative in order to induce reflexive shifts of attention. The target circle subtended half a degree of visual angle around a point that was 10 degrees away from the center of the screen. The gaze cue remained onscreen for the entire duration of the trial (800 ms).

**FIGURE 1:**
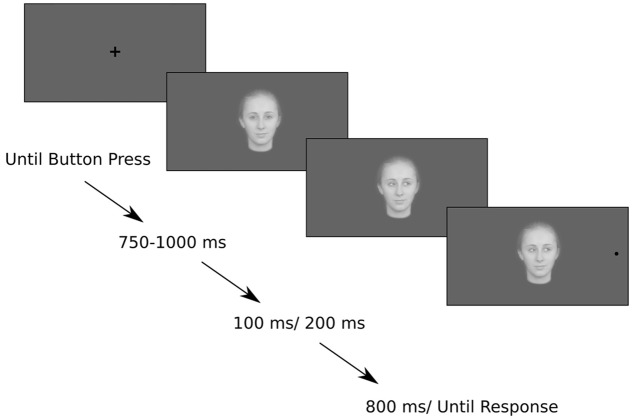
**Example of one trial in Experiment 1 with shift of the eye gaze as a valid cue**.

Participants were instructed to maintain fixation on the centrally presented face for the period when the gaze was directed toward them and to continue looking at the face when the eye gaze changed direction. They were asked to move their eyes toward the black target circle as soon as it appeared on the left or right. They were instructed to respond as fast as possible but not at the expense of accuracy (**Figure 1**). They were told that direction of the eye gaze was not informative.

#### Data Analysis

In order to calculate SRT, we analyzed the subject’s gaze position after the target was displayed on screen. We took the first time point at which the *x* coordinate of the gaze position exceeded the borders of the centrally presented face to be the SRT. All the trials landed in a neighborhood of 1 degree of visual angle around the target. We marked the trials in which eye movements were made in the direction opposite to that of the target as incorrect trials. We did not include trials in which eye movements failed to land on the target in subsequent analyses (<2% of total trials).

We also marked trials in which eye gaze did not cross the image border in either direction as errors and included them in the calculation of accuracy. Finally, we discarded the trials that represented reaction times shorter than 80 ms.

We used MATLAB for defining saccades and R (R Core Team, 2013) for subsequent statistical analysis. We constructed a linear mixed model with log transformed SRT as the dependent variable, familiarity condition, validity and the SOA as fixed effects and participants as random effects. The reaction times were log transformed in order to fit the assumptions of linear mixed models. Package lme4 from CRAN was used for mixed models analysis (Bates et al., 2014). For both logit and linear mixed models, different models with random effects were created and compared with log likelihood ratio tests; the model that yielded the lowest Akaike’s information criterion (AIC) was chosen. Once a final model was determined, statistical significance of the main and interaction effects was tested using a Type 3 Analysis of Deviance, as implemented in the package *car* (Fox and Weisberg, 2010). Note that an analysis of deviance tests the differences in deviance of a model using a chi-square test, and thus chi-square values are reported for both linear and logit models.

### Results

**FIGURE 2:**
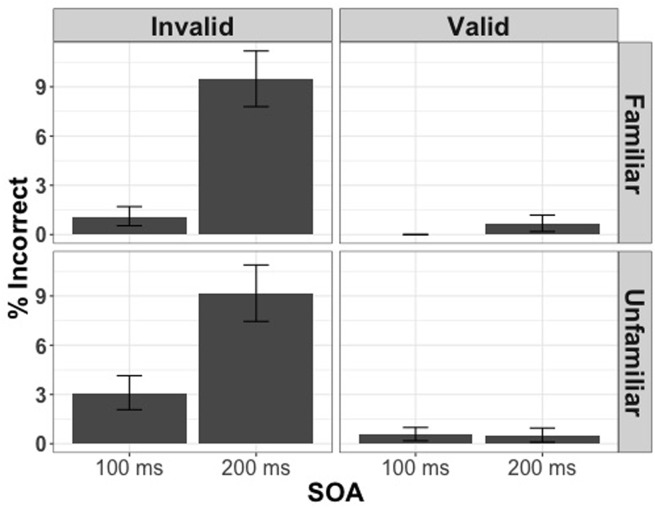
**Percentage of incorrect responses for 100 and 200 ms split by cue validity and familiarity condition in Experiment 1**.

We first analyzed the entire set of trials in order to assess if any of the experimental manipulations influenced the number of errors made throughout the course of the experiment. Using participants’ response (correct or incorrect) as the dependent variable, and familiarity condition, cue validity, and SOA as predictors, we constructed a generalized linear mixed model with binomial error distribution and logit model as linking function (lme4 package). We found that the participants made significantly more errors on incongruent trials [χ^2^(1) = 121.35, *p* < 0.001] and, surprisingly, the longer SOA [χ^2^(1) = 93.92, *p* < 0.001] but the familiarity condition did not have a statistically significant effect on the number of errors made [χ^2^(1) = 0.31, *p* = 0.72] (**Figure 2**).

**FIGURE 3:**
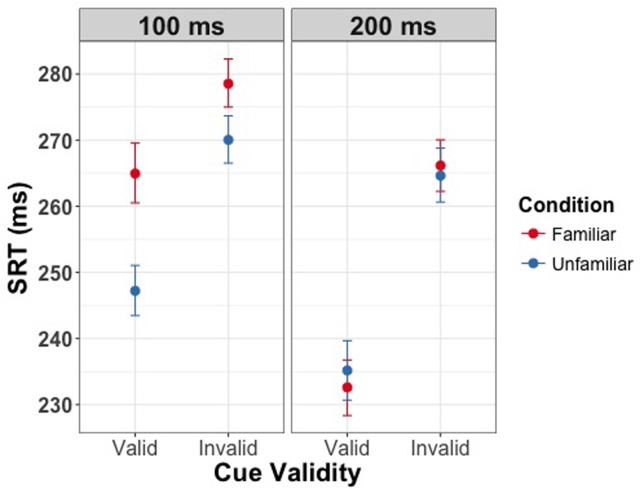
**Saccadic response time (SRT) as a function of validity of the eye gaze cue in Experiment 1**. Left panel depicts the effect at the SOA of 100 ms, right panel depicts the effect at 200 ms. Error bars represent bootstrapped 95% confidence intervals around the mean. Familiarity of the gaze cue results in longer latencies for saccadic response at an SOA of 100 ms.

We then assessed whether the three experimental manipulations influenced the SRT when responding to the presence of the target. Our results revealed significant main effects of all three experimental manipulations. Firstly, we found a main effect of cue validity [χ^2^(1) = 99.98, *p* < 0.001] showing shorter reaction times for valid as compared to invalid trials. Secondly, we found a main effect of SOA [χ^2^(1) = 83.44, *p* < 0.001] indicating slower responses for the shorter delay between the cue and target. Finally, we found a main effect of familiarity [χ^2^(1) = 9.75, *p* = 0.002] with longer reaction times for familiar faces. Moreover, we found significant interactions of familiarity condition and SOA [χ^2^(1) = 18.84, *p* < 0.001], and of cue validity and SOA [χ^2^(1) = 35.73, *p* < 0.001]. There was no significant interaction of cue validity and familiarity [χ^2^(1) = 1.06, *p* = 0.30]. The three-way interaction between validity, familiarity condition and SOA was significant [χ^2^(1) = 5.75, *p* = 0.02]. Saccades toward the target were slower on invalid trials than on valid trials, and this effect of validity was larger with the longer SOA between the cue and target. Moreover, the interaction between validity, familiarity condition, and SOA indicates that the effect of familiarity only holds if the delay between cue and target is 100 ms. Thus, at the short SOA but not the long SOA participants were slower to saccade to both valid and invalid targets and the effect of cue validity was smaller if the cue was signaled by a familiar face as compared to a stranger’s face (**Figure 3**). Effect sizes are provided in **Tables 1**–**3**.

**Table 1:** Main effect of familiarity in Experiment 1: Familiar RT – Unfamiliar RT.

SOA	Validity condition	Mean difference of familiarity (ms)	Pooled standard deviation (ms)	Cohen’s *d*	Confidence interval
100 ms	Invalid	8.42	57.2	0.15	0.06, 0.26
100 ms	Valid	17.6	63.1	0.28	0.18, 0.40
200 ms	Invalid	1.21	63.2	0.02	-0.06, 0.12
200 ms	Valid	-2.06	72	-0.03	-0.11, 0.07

**Table 2:** Main effect of cue validity in Experiment 1: Invalid RT – Valid RT.

SOA	Familiarity condition	Mean difference of cue validity (ms)	Pooled standard deviation (ms)	Cohen’s *d*	Confidence interval
100 ms	Familiar	13.8	59.0	0.23	0.14, 0.33
100 ms	Unfamiliar	23.0	57.3	0.40	0.32, 0.49
200 ms	Familiar	32.2	60.0	0.54	0.46, 0.64
200 ms	Unfamiliar	29.0	63.1	0.46	0.38, 0.56

**Table 3:** Main effect of SOA in Experiment 1: 100 ms SOA – 200 ms SOA.

Familiarity condition	Validity condition	Mean difference of SOA (ms)	Pooled standard deviation (ms)	Cohen’s *d*	Confidence interval
Familiar	Invalid	13.7	60.0	0.23	0.14, 0.35
Familiar	Valid	32.1	69.1	0.46	0.37, 0.57
Unfamiliar	Invalid	6.47	63.1	0.10	0.02, 0.20
Unfamiliar	Valid	12.5	72.0	0.17	0.09, 0.27

### Interim Discussion

We observed longer reaction times following familiar faces at the shorter delay between the cue and target onsets. These results suggest that participants are slower in looking away from familiar faces as compared to faces of strangers, thereby delaying the reaction time in response to the target both for valid and invalid gaze cues. We reasoned that the same results might not hold true if the task does not require the participant to explicitly look away from the centrally presented face. In order to test this hypothesis, we designed an experiment to test the effect familiarity of the cue on shifts of attention that do not involve saccades to the target.

## Experiment 2

In this experiment, we investigated whether findings reported in Experiment 1 would hold if the response to the attended target did not involve explicit eye movements away from the centrally presented face cue, we tested the same participants in a paradigm that involved a manual response via a button press.

### Materials and Methods

#### Participants, Stimuli, and Equipment

The stimuli and testing equipment were exactly the same as Experiment 1. Thirteen of the original 15 participants participated in this experiment (seven male, Mean age: 27.38 ± 2.06). All participants provided written informed consent to participate in the experiments, and were paid. The Dartmouth Committee for the Protection of Human Subjects approved the experiment (Protocol 21200).

#### Experimental Paradigm

The sequence for presenting stimuli within a trial was exactly the same as in Experiment 1 (see **Figure 1**), except that we did not vary the SOA in this experiment—since results of Experiment 1 indicated that the effects of interest are present in the shorter delay (100 ms) between the cue and target. Participants performed three blocks of 100 trials each, resulting in a total of 300 trials. Over the course of the experiment, the picture of each individual identity was repeated 50 times. Eye movements were recorded to ensure that participants maintain central fixation (see Experiment 1 on details how eye movements were recorded). Participants responded with their dominant hand.

The task was similar to Experiment 1, except that participants were asked to respond manually by pressing the left or right arrow key to indicate the side where the black target circle appeared. The participants were instructed to maintain fixation on the centrally presented face and only respond when the target appeared in their peripheral vision. Trials in which eye movements were made in this period were discarded (<1%). As in Experiment 1, participants were instructed to respond as fast as possible but not at the expense of accuracy.

#### Data Analysis

We discarded trials in which reaction time was less than 100 ms as anticipatory responses. Moreover, we also removed trials in which eye movements were made as they reflected the failure to maintain fixation.

We constructed a linear mixed model with log transformed manual response time as the dependent variable, the familiarity condition and cue validity as the fixed effects and the participants as random effects. We created different models with random effects and compared them with log likelihood ratio tests and chose the model that yielded the lowest Akaike’s information criterion (AIC) was chosen (see Data Analysis of Experiment 1). The values reported in the results were obtained from Type 3 Analysis of Deviance on each model, performed with the function ANOVA from package car (Fox and Weisberg, 2010).

### Results

**FIGURE 4:**
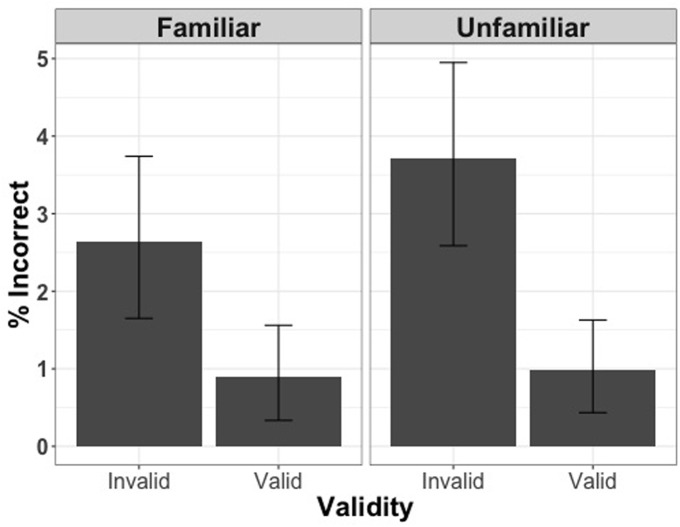
**Percentage of incorrect responses for valid and invalid trials when the face cue was familiar or unfamiliar in Experiment 2**.

Analysis of the entire set of trials (including correct and incorrect responses) with accuracy as the dependent variable revealed that more incorrect responses were made for incongruent trials [χ^2^(1) = 21.32, *p* < 0.001], but familiarity did not have an effect on the accuracy of responses [χ^2^(1) = 1.60, *p* = 0.2] (**Figure 4**).

**FIGURE 5:**
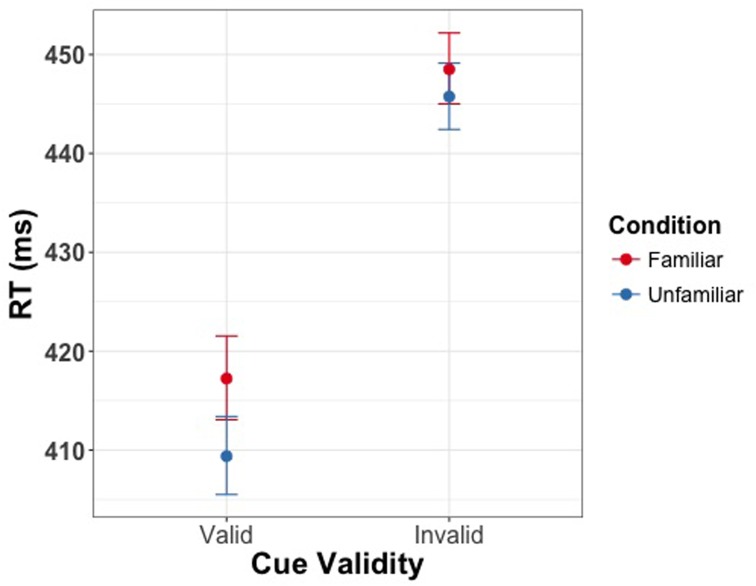
**Manual response time as a function of validity of the gaze cue in Experiment 2**. Error bars represent bootstrapped 95% confidence intervals around the mean. Participants were slower on invalid trials, and their latency was affected by the familiarity of the cues.

The linear mixed model constructed for reaction time as a function of familiarity condition and validity revealed significant main effects of cue validity [χ^2^(1) = 51.34, *p* < 0.001] and familiarity [χ^2^(1) = 7.21, *p* = 0.007] (**Figure 5**). The magnitude of the effect of familiarity in this Experiment (5.5 ms) was smaller than the effect of familiarity found at the 100 ms SOA of Experiment 1 (15 ms). Finally, as observed in Experiment 1, there was no significant interaction of cue validity and cue familiarity [χ^2^(1) = 0.98, *p* = 0.32]. Effect sizes for this experiment are reported in **Tables 4**, **5**.

**Table 4:** Main effect of familiarity in Experiment 2: Familiar RT – Unfamiliar RT.

Validity condition	Mean difference of familiarity (ms)	Pooled standard deviation (ms)	Cohen’s *d*	Confidence interval
Invalid	5.04	50.2	0.10	-0.06, 0.21
Valid	6.85	60.4	0.11	0.02, 0.23

**Table 5:** Main effect of cue validity in Experiment 2: Invalid RT – Valid RT.

Familiarity condition	Mean difference of cue validity (ms)	Pooled standard deviation (ms)	Cohen’s *d*	Confidence interval
Familiar	32.0	54.3	0.59	0.50, 0.69
Unfamiliar	33.9	50.2	0.67	0.59, 0.78

### Interim Discussion

Results from Experiment 2 revealed an effect of familiarity on attention shifting on the timescale of 100 ms, similar to what was found in Experiment 1 but with a smaller magnitude. In both experiments, reaction times to targets were slower for familiar faces. Altogether, we found effects of slowing of attentional disengagement by familiar face stimuli in both experiments, suggesting that familiar faces are highly salient stimuli that briefly hold attention, interfering with shifts of attention to other locations. In order to assess if these results arose from differences in processing the gaze cue itself in personally familiar and unfamiliar faces, we ran one more experiment. We assessed differences in processing eye gaze in familiar and unfamiliar faces in the absence of a task requiring a shift in spatial attention by asking participants to make a manual response to indicate the direction of eye gaze in familiar and unfamiliar faces.

## Experiment 3

In order to ensure that the results reported in Experiments 1 and 2 did not come from differences in processing the eye gaze from familiar and unfamiliar faces, we asked participants to indicate the direction of eye gaze changes in familiar and unfamiliar faces with a manual response.

### Materials and Methods

The stimuli and testing equipment were exactly the same as in Experiment 2.

Nine (four male, Mean age: 28.11 ± 0.56) of the original 13 participants from Experiment 2 participated in this experiment. Three of the previous participants had graduated and left the campus and one did not respond. All participants provided written informed consent to participate in the experiment, and were paid. The Dartmouth Committee for the Protection of Human Subjects approved the experiment (Protocol 21200).

#### Experimental Paradigm

The experimental paradigm was similar to Experiments 1 and 2, except that there was no target following the change in eye gaze. Participants performed three blocks of 50 trials each, resulting in a total of 150 trials. Over the course of the experiment, the picture of each individual identity was repeated 25 times.

Participants were instructed to press either the left arrow or the right arrow key to indicate the direction of the eye gaze change (either to the left or to the right) of the centrally presented face. As in the first two experiments, participants were instructed to be as fast as possible in their response.

#### Data Analysis

We rejected trials with reaction times less than 100 ms.

We constructed a linear mixed model with log transformed manual response time as the dependent variable, the familiarity condition as the fixed effect and the participants as random effects. The values reported in the results were obtained from Type 3 Analysis of Deviance on each model, performed with the function ANOVA from package car (Fox and Weisberg, 2010).

### Results

**FIGURE 6:**
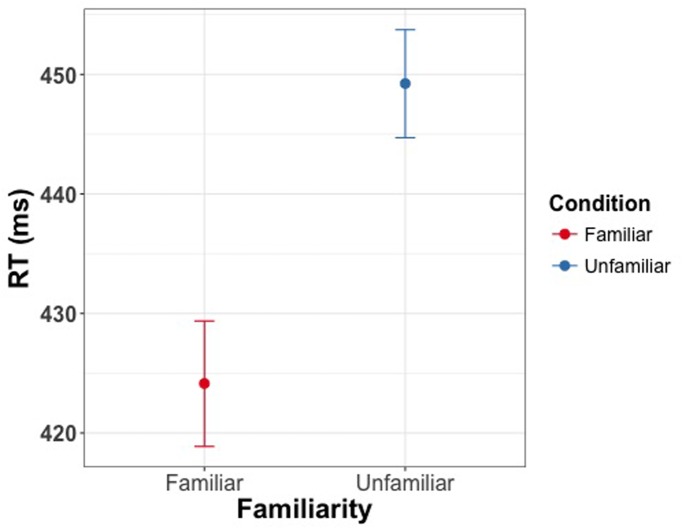
**Manual response times as a function of familiarity of the face cue in Experiment 4**. Participants were asked to indicate direction of eye gaze via button press. Participants were faster in detecting the direction of gaze for familiar faces. Error bars represent bootstrapped 95% confidence intervals around the mean.

The linear mixed model revealed a significant effect of familiarity condition on reaction time for reporting the direction of changes in eye gaze direction [χ^2^(1) = 39.75, *p* < 0.001], with shorter reaction times for familiar faces (*M* = 425 ms, CI = [420 ms, 430 ms]) than for unfamiliar faces (*M* = 450 ms, CI = [445 ms, 454 ms]) (**Figure 6**). Cohen’s *d* for this effect was 0.53. There was no effect of familiarity on accuracy [χ^2^(1) = 0.76, *p* = 0.38].

### Interim Discussion

The results of this experiment, in line with the findings of Visconti di Oleggio Castello et al. (2014), show faster processing of eye gaze in personally familiar as compared to unfamiliar faces. These results further support the hypothesis that slower response to targets with personally familiar face cues as compared to unfamiliar face cues, is due to the holding of attention by the personally familiar faces rather than to slower processing of eye gaze shifts.

## Discussion

We tested the interaction between reallocation of spatial attention mediated by shifts in eye gaze and personal familiarity with faces. We used an adapted version of the Posner paradigm to study attention shifts elicited by eye gaze movements in others. Overall, the results showed facilitation of responses to peripheral targets in trials with congruent cues as compared to trials with incongruent cues. These results replicate the effect of cue validity on saccadic response time found in prior research (Kuhn and Kingstone, 2009). Consistent with earlier studies, reaction times were faster and the cue validity effect was larger at the longer SOA of 200 ms (Friesen and Kingstone, 1998; Kuhn and Kingstone, 2009). Here, we report for the first time an interaction of SOA and personal familiarity wherein face familiarity slows the redirection of spatial attention mediated by eye gaze at short but not long SOAs. More specifically, in our first experiment we found that gaze shifts in personally familiar faces, as compared to in unfamiliar faces, elicited slower saccadic response times at a short SOA of 100 ms between the gaze shift and the onset of the peripheral target. Familiarity did not modulate the effect of cue validity. No modulation by familiarity was recorded at the longer SOA of 200 ms, indicating that the slowing of attention shifts due to cue face familiarity is brief. The results of the second experiment, which required maintaining fixation on the cue face and manual responses, showed that the effect of familiarity was not specific to saccadic responses that required looking away from the face. The size of the familiarity effect, however, was smaller than for saccadic responses. The additional time to prepare a manual response (over 150 ms) may diminish the familiarity effect, which appears to be rapidly fading.

The third experiment investigated whether the results reported in Experiments 1 and 2 were due to differences in processing of eye gaze in familiar and unfamiliar faces, rather than to differences in the holding of attention by familiar and unfamiliar faces. Results from Experiment 3 showed that personal familiarity significantly facilitated detection of the direction of gaze shifts, as compared to unfamiliar faces, indicating that the familiarity effect on gaze-cued attention shifts to the periphery, which was in the opposite direction, is not due to slower detection of the gaze cue itself. Interestingly, the facilitation of eye gaze detection by familiarity appears to be stronger for more demanding tasks. In a visual search task, we found a larger effect of facilitation on RT, over 100 ms (Visconti di Oleggio Castello et al., 2014). By contrast, we found only a non-significant trend toward facilitation in a simple gaze change detection task (see Supplementary Material) unlike the task in Experiment 3 that required indicating the gaze change direction. Therefore, despite facilitated detection of eye gaze shifts in familiar faces, reallocation of attention away from the face is slowed by personal familiarity due to slowed disengagement of attention.

To summarize, our results indicate that familiarity delays gaze-cued attentional shifts at short latencies by briefly slowing deployment of attention away from familiar faces. Our results suggest that the familiar faces capture attention (Simons, 2000), and that this effect fades quickly. Slowed disengagement of attention from the familiar faces overrides any advantage of faster detection of gaze changes in familiar, as compared to unfamiliar faces. Holding of attention by personally familiar faces also has been shown by others with a different experimental paradigm, namely visual search (Devue et al., 2009).

The relationship of social salience and bias of spatial attention has been studied using personally familiar faces but with a different degree of familiarity as compared to the personally familiar faces chosen for our experiments. In previous studies, faces of colleagues or coworkers have been used as familiar stimuli. For example, Hungr and Hunt (2012) used one’s own face and a familiar confederate –the face of the experimenter– as familiar faces, and Deaner et al. (2007) used faces of professors, postdoctoral researchers and graduate students from the same department of the participants. Hungr and Hunt (2012) reported faster reaction times in the gaze cueing paradigm for the confederate’s faces, and longer for one’s own face and for faces of strangers. Deaner et al. (2007) reported a complex interaction for gender and familiarity with faster reaction times in women when participants were cued by familiar faces. In our experiments, we aimed to study the effect of familiarity that is characterized by a personal, close relationship with repeated social interactions over time rather than simple prior visual exposure. Personally familiar individuals with whom we have a close relationship have a special status and are processed more efficiently as compared to other type of familiar faces, such as famous faces and visually familiar faces. Therefore, we chose as stimuli, the faces of friends with whom the participants had a good close relationship for at least a year.

Our results provide evidence for slowed disengagement of attention from personally familiar faces, a highly salient social stimulus. The effect of slowed disengagement of attention was only found for familiar faces at a short delay between the cue and the target. Interestingly, studies of blocking by salient cues in associative learning (Denton and Kruschke, 2006; Le Pelley et al., 2014) and attentional capture by cues of high valence (Hickey et al., 2010; Anderson et al., 2011) lend support to the idea that a centrally presented cue that is highly salient, as compared to cues of less salience, leads to slower redirection of attention rather than serving as a more informative cue. Our results are consistent with these studies that used cues other than faces. Repeated exposure to the faces of familiar individuals and the semantic and emotional information associated with these identities make them socially salient. In our study, we demonstrate that this highly salient social cue holds attention rather than facilitates redirection of attention.

## Author Contributions

MIG conceived the idea for the study. VC, MIG, and AS designed the experiment. VC collected and analyzed the data. MVdOC provided suggestions for data analysis. VC prepared the manuscript, MIG, MVdOC, and AS provided critical inputs to the final version of the manuscript.

## Conflict of Interest Statement

The authors declare that the research was conducted in the absence of any commercial or financial relationships that could be construed as a potential conflict of interest.
