# Supplementary material for: Social Saliency of the Cue Slows Attention Shifts
Source: Front Psychol. 2017 May 15;8:738. doi: 10.3389/fpsyg.2017.00738 (PMC5430048; doi:10.3389/fpsyg.2017.00738)
Supplement: Supplementary file 1 [file Presentation_1.PDF]

Additional Control Experiment

Experiment 3 showed that the results reported in Experiments 1 and 2 were not due to differences in processing the eye gaze of familiar and unfamiliar faces. In that experiment we had subjects detect the direction of gaze shifts with a choice reaction time task. In an additional control experiment, we had subjects detect gaze shifts with a simple reaction time task that did not require judgment of the direction of the shift. We asked the same group of subjects that participated in Experiment 2 to respond with a button press as soon as they detected a change in the gaze direction of the foveated face.

Materials and Methods

The subjects, stimuli and testing equipment were exactly the same as in Experiments 2.

Experimental Paradigm

The experimental paradigm was similar to Experiments 1 and 2, except that there was no target following the change in eye gaze. Subjects performed three blocks of 50 trials each, resulting in a total of 150 trials. Each stimulus identity was repeated 9 times.

Subjects were instructed to press the spacebar as soon as they detected a change in the direction of eye gaze (from direct gaze to gaze to the left or right) of the centrally presented face. As in the first two experiments, subjects were instructed to be as fast as possible in their response.

Data Analysis

We rejected trials in which the reaction time was less than 100 ms on the grounds that they were likely to represent an anticipatory motor response.

We constructed a linear mixed model with log transformed manual response time as the dependent variable, the familiarity condition as the fixed effect and the subjects as random effects. The values reported in the results were obtained from Type 3 Analysis of Deviance on each model, performed with the function ANOVA from package car (Fox, Friendly & Weisberg, 2010).

Results

The linear mixed model revealed that there was no significant effect of familiarity condition on reaction time for reporting change in eye gaze ($\chi2(1) = 3.17$, $p = 0.08$). The reaction times to changes in eye gaze showed a trend toward faster detection for familiar than unfamiliar faces that did not reach statistical significance (M = 360 ms, CI = [355 ms, 365 ms] and M = 363 ms, CI = [358 ms, 368 ms], respectively).

Discussion

This experiment was conducted to assess the speed of response to eye change in familiar and unfamiliar faces with a simple gaze shift detection task.

We found a small, statistically nonsignificant decrease in the reaction time to gaze shifts in familiar as compared to unfamiliar faces. In previous work using a visual search paradigm (Visconti di Oleggio Castello et al. 2014) we showed that eye gaze direction is processed faster in familiar faces, and Experiment 3 corroborated our previous results with a significant familiarity-related facilitation in a choice reaction time task for detecting the direction in gaze shifts, albeit with an effect size (25 ms) that was smaller than the effect in our earlier study (>100 ms). The advantage of familiarity might have been attenuated by the additional time required for manual responses as compared to saccadic responses. In this control experiment, the effect was even smaller (3 ms) with a simpler detection task, suggesting that the facilitation by familiarity is greater for more demanding tasks.
